# Vertical integration of microchips by magnetic assembly and edge wire bonding

**DOI:** 10.1038/s41378-019-0126-6

**Published:** 2020-02-24

**Authors:** Federico Ribet, Xiaojing Wang, Miku Laakso, Simone Pagliano, Frank Niklaus, Niclas Roxhed, Göran Stemme

**Affiliations:** Division of Micro and Nanosystems, School of Electrical Engineering and Computer Science, KTH Royal Institute of Technology, Malvinas väg 10, 10044 Stockholm, Sweden

**Keywords:** Nanoscience and technology, Electrical and electronic engineering

## Abstract

The out-of-plane integration of microfabricated planar microchips into functional three-dimensional (3D) devices is a challenge in various emerging MEMS applications such as advanced biosensors and flow sensors. However, no conventional approach currently provides a versatile solution to vertically assemble sensitive or fragile microchips into a separate receiving substrate and to create electrical connections. In this study, we present a method to realize vertical magnetic-field-assisted assembly of discrete silicon microchips into a target receiving substrate and subsequent electrical contacting of the microchips by edge wire bonding, to create interconnections between the receiving substrate and the vertically oriented microchips. Vertical assembly is achieved by combining carefully designed microchip geometries for shape matching and striped patterns of the ferromagnetic material (nickel) on the backside of the microchips, enabling controlled vertical lifting directionality independently of the microchip’s aspect ratio. To form electrical connections between the receiving substrate and a vertically assembled microchip, featuring standard metallic contact electrodes only on its frontside, an edge wire bonding process was developed to realize ball bonds on the top sidewall of the vertically placed microchip. The top sidewall features silicon trenches in correspondence to the frontside electrodes, which induce deformation of the free air balls and result in both mechanical ball bond fixation and around-the-edge metallic connections. The edge wire bonds are realized at room temperature and show minimal contact resistance (<0.2 Ω) and excellent mechanical robustness (>168 mN in pull tests). In our approach, the microchips and the receiving substrate are independently manufactured using standard silicon micromachining processes and materials, with a subsequent heterogeneous integration of the components. Thus, this integration technology potentially enables emerging MEMS applications that require 3D out-of-plane assembly of microchips.

## Hands-off production of 3D sensing microsystems

A strategy for the magnetically assisted vertical assembly of microchips into their substrates and for their electrical connection could offer a versatile solution for the construction of fragile and complex miniaturized sensors. Conventional contact-based assembly methods such as pick-and-place manipulation have the potential to damage these sensitive parts, while existing contactless approaches offer poor control for high-volume manufacturing. Researchers led by Xiaojing Wang and Federico Ribet at the KTH Royal Institute of Technology in Sweden have now devised a manufacturing method that enables efficient construction of such devices. Their technique relies on matched microchip-substrate geometries, with patterned ferromagnetic material to guide assembly, and wire bonding to establish interconnections. This manufacturing process can be performed under conventional conditions at room temperature and should offer a versatile and robust means for producing 3D microelectromechanical system (MEMS) components.

## Introduction

Silicon microfabrication techniques are well-established, scalable, and cost-efficient. However, they typically do not allow the creation of complex three-dimensional (3D) structures. At the same time, many emerging microelectromechanical system (MEMS) applications such as inductors^1^, radio frequency antennas^2^, optical attenuators^3^, flow sensors^4^, and biosensors^5^ require vertical out-of-plane structures with complex 3D geometries to fulfill their function. Therefore, the assembly and electrical packaging of micromachined in-plane structures into out-of-plane functional devices has become a need, and a challenge, for MEMS integration^6^.

Various methods for realizing 3D assembly of micro-components have been reported and can be generally divided into contact-based and contactless manipulation approaches. Contact-based assembly methods typically comprise two categories: (I) serial pick-and-place manipulation using micro-grippers or probes^7–11^ and (II) parallel transfer printing^12,13^. The former can be used for handling objects with dimensions in the millimeter to nanometer range, but suffers from efficiency and scalability, whereas the latter typically do not allow vertical lifting of the transferred components. In addition, contact-based methods pose challenges in the assembly of fragile micro-components, unlike contactless methods, which are by nature more compatible with fragile devices. Different principles have been explored to address the contactless out-of-plane in-situ manipulation of planar structures attached to a substrate, including magnetically induced bending^1–4,14,15^, bimorph-based bending by differential internal stress^16,17^ or thermal stress^18,19^, surface tension-driven assembly^20–22^, and other more complex approaches^8,23^. However, most of these techniques are not versatile methods to assemble microchips in a separately fabricated receiving substrate, which might be needed in, e.g., sensing applications^5^. Besides, these methods typically either require the use of unconventional materials or high temperatures, or do not involve the formation of electrical connections. Other approaches for out-of-plane 3D assembly of structures without direct contact utilize stochastic self-assembly principles to place discrete components on a surface or into a template of holes, based on magnetic attraction^24–26^, dynamic force fields provided by gravitational and magnetic forces^27^, and controlled vibration stimuli^28^. In particular, to perform out-of-plane assembly of discrete components into receiving holes, vertical insertion of components into through-silicon trenches using magnetic assembly has been proposed^29^, as well as magnetic assembly of ferromagnetic metallic wires for through-substrate vias^30–32^. The use of magnetic assembly is of particular interest for microchip handling, because it allows non-contact manipulation of microchips. However, the previously proposed solutions either do not allow vertical assembly of microchips or were only applied to natively ferromagnetic components. Moreover, the vertical orientation of the microchip assembly, often critical to the proper functioning of the device, could not be controlled using these methods, so that the devices could be assembled into the receiving holes without control of the resulting orientation.

The above-mentioned limitations regarding spatial and rotational control of the microchips also limited the possible alternatives to electrically contact the devices. In state-of-the-art solutions, e.g., the contacts to the devices were made on both the frontside and backside of the receiving substrate by metallization, to contact the components that were embedded inside the through-substrate holes^22,23^. However, these approaches are limited to devices with a maximum of two inter-changeable contacts such as resistors or capacitors. Moreover, these contacting methods involved harsh processing steps, including high temperatures, physical polishing, chemical exposure (polymers and solvents), and plasma etching, which are incompatible with, e.g., biosensors. To realize large-scale and versatile electrical contacting of heterogeneously assembled microchips, it is preferable to use a well-established and high-throughput industrial technique such as wire bonding. In fact, despite being a serial process, wire bonding is commonly used for mass production, with a potential speed of ~200 bonds per minute, simultaneously enabling versatility due to the pattern recognition features integrated in the wire bonding process^33^. To allow attachment of the wire bonds, in-plane metallic pads are typically required. However, wire bonding has also been used for more unconventional applications^34^ including direct wire bonding on in-plane-patterned holes in silicon^35^.

No conventional approach currently offers such a versatile solution to vertically assemble sensitive or fragile microchips into a separate receiving substrate and to create electrical connections. In particular, a challenge that still remains unsolved is the creation of a volume-manufacturable method for directional vertical 3D assembly of discrete, fragile, and sensitive silicon-based microchips and their electrical connection to the receiving substrate, using tools and micromachining methods that do not adversely affect the functionality, integrity, or fabrication complexity of the produced devices.

In this study, we present a novel heterogeneous integration approach for vertical directional assembly and interconnection of conventionally micromachined planar silicon microchips in a receiving substrate. This method combines the principle of magnetic assembly^29,30^, to realize out-of-plane insertion of microchips into matching holes in the receiving substrate, and room-temperature wire bonding on the top sidewalls of the vertically assembled microchips, to achieve electrical interconnections between the frontside planar electrodes of the microchips and the bond pads on the receiving substrate. We demonstrate that our approach provides controlled vertical orientation of the assembled microchips, due to a combination of shape matching and a striped patterning of a ferromagnetic coating on the device backside, and allows the creation of protruding 3D structures, which have not previously been realized using conventional magnetic-field-assisted micro-manipulation approaches^24–27,29^. The assembled microchips are then electrically connected to the receiving substrate through a new edge wire bonding process, all without involving harsh processing, high temperatures, or exotic materials, while taking advantage of the extremely mature and low-cost wire bonding infrastructure. To our knowledge, no previous solution has addressed contacting of vertically placed microchips with such requirements. This method thus enables the heterogeneous vertical assembly and contacting of silicon microchips for a wide range of applications, including advanced 3D sensing devices with integrated biosensors and other sensitive and fragile components.

## Results and discussion

### Microchip design and vertical integration method

**Fig. 1:**
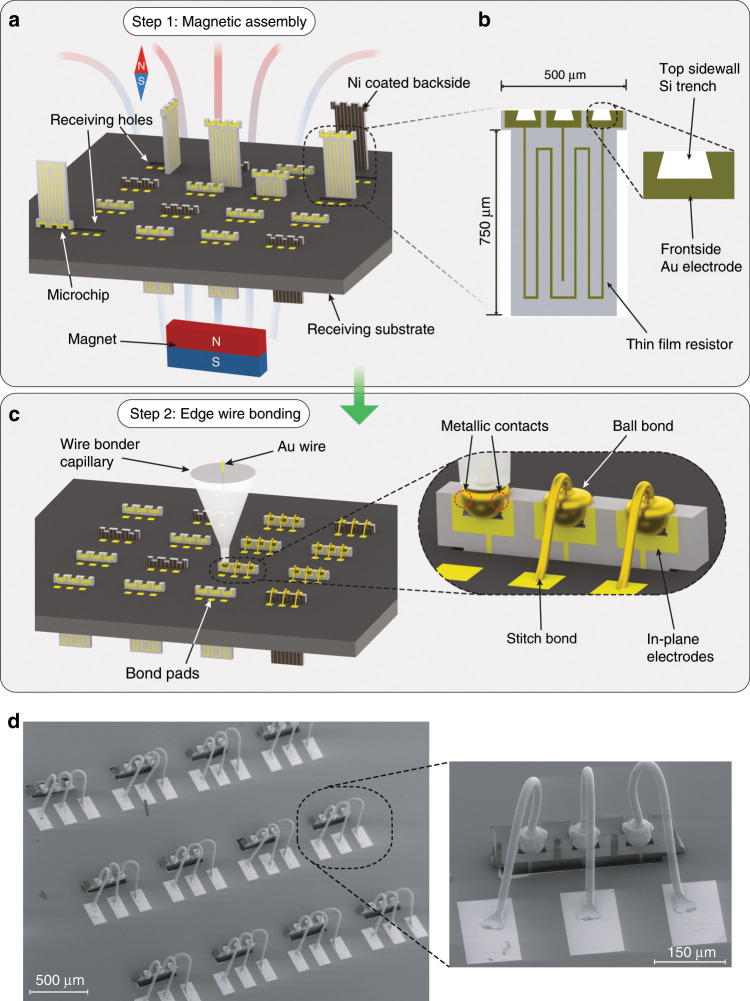
Method for the vertical integration of microchips and final result. **a** Illustration of the vertical magnetic assembly process. The microchips have a patterned nickel coating on the backside and are spread on a receiving substrate with matching through-holes. Upon the presence of a magnetic field, the microchips lift vertically and, by moving the magnetic field, they move accordingly until they fall inside the holes. **b** Illustration of the microchip design. The microchips feature three frontside contact electrodes and a thin-film resistor connecting the rightmost and leftmost electrodes. The top sidewall trenches constitute the receiving structures for the ball bonds. The “T”-shaped design prevents the microchips to enter the holes upside down and causes the microchips to rest on the through-holes once in position. **c** Illustration of the edge wire bonding process to realize the electrical contacting of the previously assembled microchips. Inset: deformed Au free air balls (FABs) create metallic interconnections between the frontside electrodes of the microchips and the receiving substrate. **d** SEM image of a wire-bonded microchip array. Here, the microchips were manually oriented and assembled to face the same direction, purely for experimental and aesthetic reasons.

To verify the viability of our vertical assembly and contacting method, we realized demonstrators consisting of arrays of 4 × 4 microchips, as illustrated in Fig. 1a, c. The method comprises two steps: (I) magnetic assembly of discrete microchips into rectangular through-holes in the receiving substrate (Fig. 1a, b) and (II) subsequent edge wire bonding to create reliable chip-to-substrate electrical interconnections (Fig. 1c). A portion of a finalized array is shown in Fig. 1d. Using this method, the receiving substrate and the microchips can be separately fabricated using standard in-plane silicon microfabrication technologies and be subsequently integrated.

To perform the magnetic assembly, the microchips were first released and collected from the source wafer and subsequently placed on top of the receiving substrate. The receiving substrate contained receiving holes where the microchips were to be assembled and also the bond pads for electrical connection. A thin nickel layer deposited on the backside of the microchips made the microchips reactive to an external magnetic field, enabling magnetic-field-assisted control. The microchips were manipulated, without direct contact, using a permanent magnet placed below the receiving substrate. For the successful magnetic assembly of the microchips, their lifting direction in the external magnetic field had to be controlled. This was achieved by patterning the nickel layer into narrow stripes, which induced preferential vertical lifting of the microchips along a defined axis, as described in detail in the next section. The presence of the magnetic field thus resulted in the vertical lifting of the microchips and then, upon lateral motion of the magnet, the microchips followed the dynamic magnetic field until they were eventually inserted into the receiving holes.

The specific “T”-shape of the microchips was designed to prevent the microchips that lifted upside down from being assembled into the receiving holes with a wrong orientation. The designed “T”-shape of the microchips comprised a main rectangular body and a slightly wider overhanging top (Fig. 1b). The overhanging top of the microchips is wider than the receiving holes and thus prevented the microchips to enter the receiving holes in this direction. The realization of the “T”-shaped microchips consumes additional wafer area compared with rectangular microchips. In general, the larger the microchip size, the smaller in proportion is the additional silicon area required by the overhanging top part of the “T”-shape. For the microchips with dimensions presented in Fig. 1a, the additional microchip area in comparison with a purely rectangular chip shape is of the order of 10%. Importantly, in the presented process, the creation of the “T”-shape does not add any manufacturing steps in comparison with the rectangular chip shape and thus the “T”-shaped chips do not increase the manufacturing complexity of the system. The rotational position around the vertical axis of the microchips once assembled is defined by the shape of the holes in the receiving substrate. In the case of rectangular holes, the orientation of the microchips around this axis is limited to two possible directions, with either their frontside or backside facing the bond pads on the receiving substrate (Fig. 1a). In the case of circular receiving holes, no control of the position around this axis of rotation is achieved, as shown in Supplementary Video 4. In both situations, the proposed electrical contacting process can adapt to the rotational variability, as discussed in more detail below.

To realize electrical connections between the vertically placed microchips and the receiving substrate, a new edge wire bonding process was developed. To perform this process, standard gold (Au) wires and ball-stitch bonding loops were used, after fixation of the hanging part of the microchips by glue droplets. The microchips were designed with standard metal electrodes on their frontside, as in common planar microfabrication processes, and etched Si trenches on their top sidewalls to mechanically fixate the Au ball bonds (Fig. 1b, inset). The etched Si trenches were oxidized before wire bonding to guarantee electrical insulation between the different interconnections. The diameter of the Au free air ball (FAB) generated for the ball bond was larger than the opening of the sidewall trench, thus inducing plastic deformation of the FAB during the compression bonding process. The plastic deformation of the FABs inside the trenches and around the edges of the microchips results in two different beneficial effects. First, the deformed FABs contact the frontside electrodes, creating metallic wire bond interconnections from the frontside electrodes of the microchips to the bond pads on the receiving substrate. Second, the deformed bottom parts of the FABs wedge into the tapered sidewalls of the trenches, providing fixation of the ball bonds and, thus, mechanical stability and robustness to the bonds. The geometries of the sidewall trenches of the microchips are critical to obtain reliable metallic contacts and mechanically stable ball bonds during the edge wire bonding procedure (Supplementary Fig. S1 and Supplementary Table S1). In particular, the geometry shown in the inset of Fig. 1b is based on a trapezoid trench. A suitable combination of trench width, depth, and angle can help achieve sufficient plastic deformation of FABs, so that both electrical contact with the frontside electrodes and wedging inside the trench for mechanical fixation can be simultaneously achieved. As illustrated in Fig. 1b, the microchips feature three frontside electrodes. Although the microchips consist of simple thin-film resistors to provide a characterization platform to test the quality and reliability of the connections, the presented method can be applied to various devices depending on the targeted application, by adding the described necessary features during the microfabrication process. Finally, this method can be of use also for small-scale or even single microchip assembly, when the size and fragility of the chips entail challenging handling and electrical contacting.

### Magnetic assembly

**Fig. 2:**
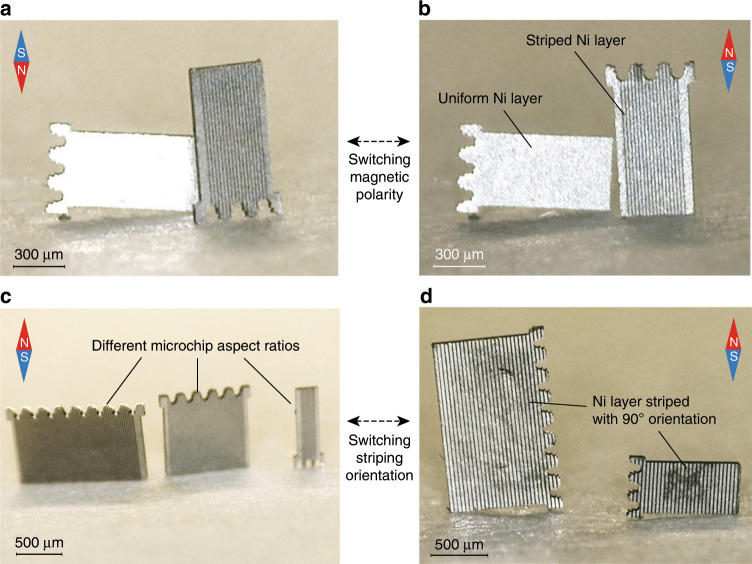
Controlled microchip lifting for magnetic assembly. **a**, **b** Microscope images showing controlled vertical lifting directionality of the microchips (500 µm-wide) in the presence of a magnetic field due to the striped nickel coating on the backside. In contrast, a microchip with a uniform nickel layer can lift in the wrong direction, along its long edge. By changing the polarity of the magnet, the microchips can be flipped upside down. **c**, **d** The lifting direction of the microchips was controlled by striping of the nickel coating. The resulting lifting direction is independent of the microchips’ aspect ratios (length/width), ranging from 2.3 to 0.5. Changing the striping direction by 90° caused a matching change of the lifting direction of the microchips.

Nickel thin films are magnetically anisotropic. As a consequence, the nickel-coated microchips, upon the presence of an external magnetic field, are subject to a magnetic torque^36^. In particular, their easy axis of magnetization, i.e., the energetically favorable direction of spontaneous magnetization, lies along the film plane, whereas their hard axis of magnetization is perpendicular to the film plane^37^. Hence, when the external magnetic field generated by a permanent magnet is applied perpendicularly to the receiving substrate and the microchips are lying on the substrate surface, the magnetization of the nickel films does not align to the direction of the external field. This results in a magnetic torque that causes the microchips to lift up in a standing position, so that both nickel magnetization and external magnetic field are aligned along the film plane (Fig. 2a, b). The magnetic torque that is lifting the microchips is proportional to both the magnetic moment of the magnetized nickel film and the strength of the external magnetic field^36^, and to lift the microchips the torque needs to overcome both gravitation and stiction forces, which keep the microchips in a lying-down position. The nickel layer thickness (3 µm) and the strength of the permanent magnet used in our study were sufficient to guarantee successful lifting and motion control of the microchips. Even though lifting was achieved, a plane nickel film alone does not ensure the lifting of the microchips along a specific predetermined direction (Fig. 2a, b).

To induce a preferential lifting direction of the microchips along a predetermined axis, the nickel layer was patterned into narrow stripes. Striping enforces the easy axis of magnetization along the striping direction^38,39^. This effect is stronger with stripes that have a high aspect ratio^40^. At the same time, very dense striping results in removal of large volume of nickel, which can hinder lifting of the microchips. We used a striping geometry that balanced these two aspects, achieving reliable lifting of the microchips along the striping direction (Fig. 2c, d and Supplementary Video 1). This approach worked for a range of microchip sizes and aspect ratios (Fig. 2c, d). Even though striping causes magnetization and thus lifting along the striping axis, the magnetization can be aligned to both directions along this axis (Fig. 2a–c). This degree of freedom causes roughly half of the microchips to lift upside down when they are first introduced to an external magnetic field (Fig. 2c and Supplementary Video 1). We hypothesize that the initial magnetization and lifting direction is a direct result of the orientation of the microchips in relation to the external field when the magnetization of the nickel film happens. If the direction of the external magnetic field is flipped, the already magnetized microchips also tend to flip their lifting direction (Fig. 2a, b and Supplementary Video 4).

The microchips were moved across the surface of the receiving substrate by a magnetic force created by moving a permanent magnet horizontally below the substrate. The gradient of the external magnetic field created a force that was pulling the microchips towards the magnet^36^. The horizontal component of this pulling force was created by the magnetic field gradient at the trailing edge of the magnet and had to overcome the static friction keeping the microchips in place. The intensity of static friction depends on the coefficient of friction between the microchips and the substrate surface, and on the forces pulling the microchips onto the substrate surface, i.e., the vertical component of the magnetic pulling force and the gravitational force. Keeping both the microchips and the receiving substrate clean from contaminations was observed to improve the movement of the microchips, which we attribute to the coefficient of friction being lower with cleaner surfaces.

**Fig. 3:**
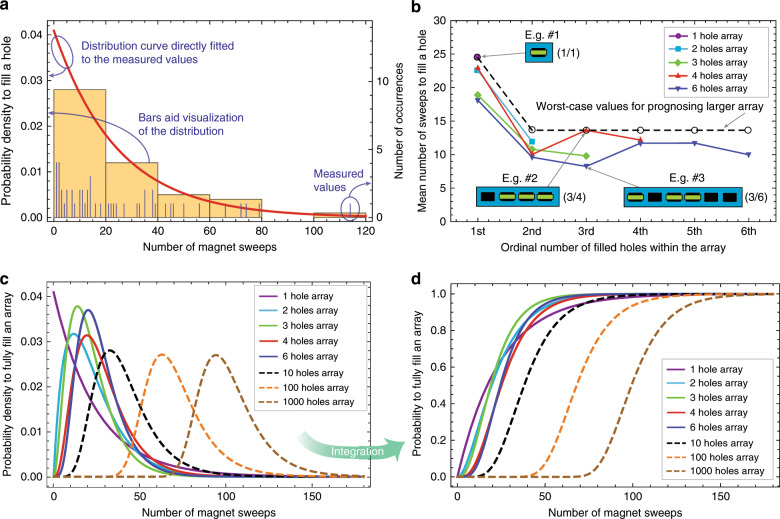
Statistical analysis of the magnetic assembly process. **a** The number of magnet sweeps needed to fill a single-hole array. Each of the 50 magnetic assembly events is represented by a blue vertical line above the horizontal axis. If the same number of sweeps was required on multiple occasions, the height of the line was increased, which is represented on the vertical axis on the right side of the figure. The exponential probability-density-distribution curve from Eq. (1) was fitted directly to the measured data points. The histogram aids the visualization of the distribution of the measured values. **b** Mean number of magnet sweeps to fill a hole in an array, as the assembly progresses, for different array sizes. Each curve shows the mean values of the exponential distributions of each consecutive hole-filling step. The mean values are scaled with the number of empty holes left in the array. The dashed line shows the worst-case measured values used for making prognosis for larger arrays. **c** Probability density distribution of completely filling an array for different array sizes. The probability densities are modeled as hypoexponential distributions whose distribution parameters are the mean values from sub-figure “b.” **d** Probability distributions of completely filling an array for different array sizes. Probability curves in this sub-figure are obtained by integrating the probability densities from sub-figure “c.” For color codes, please refer to the online version.

Magnetic assembly can be seen as a stochastic process. To investigate its behavior, we performed statistical analysis on the assembly efficiency. For these experiments, 5 different sizes of receiving-hole arrays, with up to 6 holes placed in line (see examples in Fig. 3b), were magnetically filled in 50 separate experiments. In each experiment, we used 39 microchips (size specifications in Fig. 1b), which is a significantly larger number with respect to the number of holes. This was done to guarantee a high and constant efficiency throughout the assembly process (Section 2.4, Supplementary Information). The permanent magnet was manually moved back and forth along the long edges of the rectangular holes. This movement direction allowed disassembly of chips that had not completely slid into the holes, e.g., due to being partially assembled with a wrong orientation (Supplementary Video 2). The number of movements of the magnet from one side of an array to the other (hereafter called magnet sweep) needed to fill each hole in an array was recorded. We found that the probability density of filling a single-hole array is exponentially distributed (Fig. 3a). The same distribution has been reported for other self-assembly processes^29,41,42^. When assembling larger arrays, each progressive step of successfully filling a hole in an array follows an exponential distribution (Supplementary Fig. S3). The mean number of magnet sweeps needed for each successful single-hole assembly event, scaled with the number of remaining empty holes, was larger for the first assembly event for all the array sizes. However, the mean then converges to a stable lower value (Fig. 3b). This was due to the fact that the number of chips was significantly larger than the number of holes to be filled, thereby keeping the probability of an assembly event constant throughout the assembly process. The difference between the first assembly event and the rest of the events is explained by the increased number of microchip rotations caused by the protruding top parts of already assembled microchips (Supplementary Video 2).

The magnetic assembly process can be scaled to large arrays without prohibitive time delays. The mean numbers of magnet sweeps reported in Fig. 3b were used as fitting parameters for hypoexponential distributions that describe the probability densities of completely filling arrays of different sizes (Fig. 3c), in contrast to filling just a single hole in an array. The modes of the probability densities move right with increasing array sizes (Supplementary Fig. S4f). The hypoexponential distributions together with the experimentally observed completion times for all the array sizes are presented in Supplementary Fig. S4. Hypoexponential distributions can also be used for creating forecasts for the assembly of even larger arrays. A worst-case scenario with the largest mean number of magnet sweeps observed in the experiments (Fig. 3b, dashed line) was selected for making the forecasts. The forecasted probabilities of achieving a completely filled array asymptotically approach 100% after a relatively low number of magnet sweeps, even for larger arrays. For example, an array of 1000 holes would be completely filled with 99.8% probability after only 180 magnet sweeps (Fig. 3d). To guarantee statistical consistency, due to the manual nature of the magnet motion, measurements are provided in terms of number of magnet sweeps instead of time. In our experiments, each manual sweep took a maximum of 2 s. For example, on average, an array of six holes could thus be assembled within ~2.5 min. The assembly speed can potentially be further increased by using a robotic assembly setup and by using multiple magnets in parallel for larger-scale assembly of several arrays in parallel^41^. Each magnet would be responsible to move a separate group of microchips on the surface, thus enabling parallelization of the assembly process without substantial increase of the assembly time. Moreover, the process reported here is stochastic, i.e., no active aid was used unlike in previous studies in which, e.g., a software was responsible to address the chips to the remaining empty holes and improve the resulting assembly efficiency^29^.

The assembly efficiency is affected by the range of possible rotational positions in which the microchip can fall into the receiving holes. This rotational freedom can be defined as the range of angles that the microchip can have, while still fitting into the receiving hole, as a result of the relative size of the microchip in comparison with the hole. As a consequence, and as expected, narrower microchips and larger gaps were shown to improve the assembly efficiency (Supplementary Fig. S5). In particular, in the case of a circular hole, the mean number of sweeps to fill the hole would be drastically reduced to between 1.6 and 1.9 sweeps, which correlates well with earlier results obtained when assembling circular nickel rods into circular holes^41^. Notably, in this situation the expected time for filling an array of six holes would potentially drop to only 4 s. Supplementary Video 4 shows an example of real-time assembly of microchips into circular holes. Faster assembly into circular holes might be preferable in certain applications and it would be compatible with the electrical contacting approach presented hereafter. Previous studies on assembly of micro- and nano-components showed also a positive contribution to the assembly efficiency from the use of funneled receiving holes or template aids^28,43^. In the presented situation, although these assembly-enhancing strategies could still be beneficial, they are fundamentally less applicable because of the type of motion of the vertically standing microchips and, especially, because of the need for a sufficient mechanical contact area between the hanging part of the microchip and the receiving substrate to sustain the wire bonding process.

In addition, during the assembly studies, it was observed that the width of the microchips influenced the horizontal movement of the microchips on the receiving substrate. Narrower microchips tend to move more abruptly than wider microchips, through series of jumps, rather than smoothly following the movement of the magnet (compare Supplementary Videos 2 and 4). This indicates that the horizontal pulling force experienced by wider microchips, caused by the larger volume of ferromagnetic nickel, increases more than the friction, which is increased by the larger gravitational force and the vertical component of the magnetic pulling force. The abrupt movements of the smallest microchips, such as the microprobes described later, can be reduced by vibrating the receiving substrate during assembly (Supplementary Video 4). Both vibration^26^ and movement in a liquid medium^44^ are often used to assist the assembly of micro-components, but the use of liquids would limit the potential applications of our approach and thus was not selected. Vibration was not used in any other assembly experiment than in Supplementary Video 4.

### Electrical contact quality and robustness

**Fig. 4:**
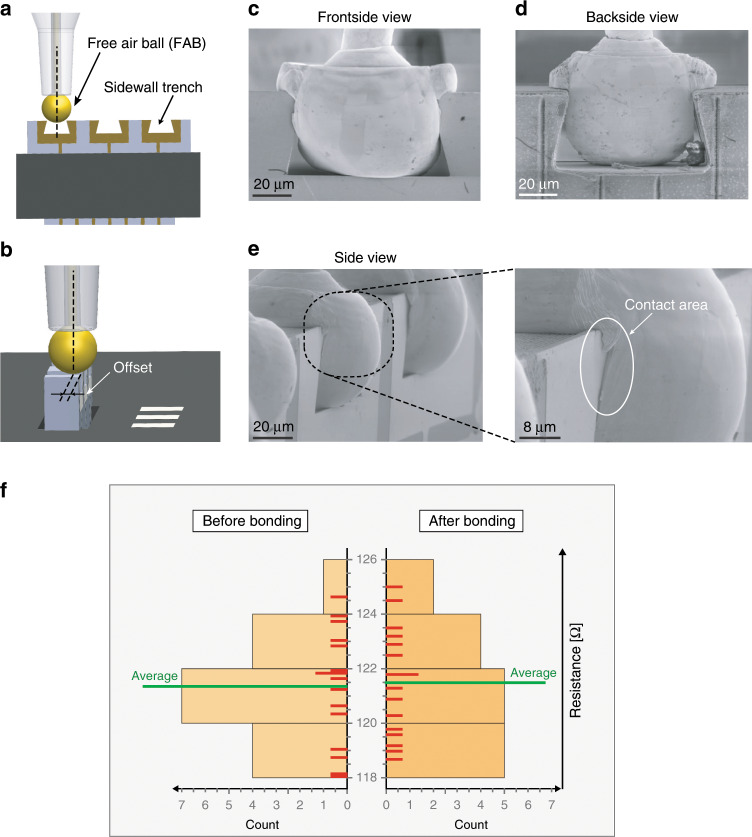
Edge wire bonding technique and results. **a**, **b** Ball bonding to the sidewall Si trenches. To increase the around-the-edge Au-to-Au contact area, the bond is performed with a certain offset (10–20 µm) on the vertically standing microchip. **c** SEM view of the microchip frontside showing the Au-to-Au contact area between the deformed Au ball and the electrode. **d** SEM image of the microchip backside showing a deformed Au ball that is partially wedged inside the trench, providing mechanical fixation of the ball bond. **e** SEM side and close-up view (inset) at the around-the-edge Au-to-Au contact area. **f** Histogram of the measured resistance of the thin-film resistors before and after wire bonding, respectively. The red lines along the *y* axis represent single resistance measurements. The mean value is shifted by only 0.19 Ω after bonding. The SEs of the sample mean before and after wire bonding are 0.52 Ω and 0.50 Ω, respectively, making the measured contact resistance of <0.19 Ω negligible.

To evaluate the quality and reliability of the contacting process, we performed wire bonding on 4 × 4 microchip arrays, resulting in completely vertically assembled and electrically connected arrays of microchips, as shown in Fig. 1d. The ball bonds were performed on the sidewall trenches of the microchips, with a certain offset in the direction of the frontside Au electrodes (Fig. 4a, b). Such a placement provided more Au contact area between the deformed FABs and the frontside electrodes. The bonding force used for forming the ball bond ranged from 400 mN to 750 mN, which was optimized to induce sufficient deformation of the FABs (90 µm diameter) and, at the same time, avoid damage to the vertically assembled microchips. Figures 4c–e show close-up scanning electron microscopic images from different angles of the ball bonds on the sidewall trenches, demonstrating clear plastic deformation of the FABs and the resulting around-the-edge metallic contacts and mechanical fixation due to wedging effects. As the microchips can enter the receiving holes with either their backside or frontside facing the bond pads on the receiving substrate, selective interconnections had to be formed. This could be readily automatized by creating asymmetrical patterns (e.g., asymmetrical spacing between the sidewall trenches) and utilizing the built-in pattern recognition functions featured by standard wire bonders. This possibility applies also to the situation in which circular receiving holes are used, with the microchips being assembled with full rotational freedom around their vertical axis. Importantly, all the bonding experiments were performed close to room temperature (30 °C) and, thus, the proposed edge wire bonding process can be used for temperature-sensitive microchips.

To evaluate the contact quality between the deformed Au balls and the frontside electrodes, the resistance values through the thin-film resistors on the microchips were measured, before and after wire bonding. The initial thin-film resistance values were measured by probing the microchip frontside electrodes directly, whereas the final resistances after bonding were measured by probing the bond pads on the receiving substrate. The measured resistance increase can thus be used to quantify the worst-case contact resistance. In particular, the main expected sources for this increase are as follows: (I) the resistance of the gold wire, calculated to be ~0.04 Ω, (II) the contact resistance between the frontside electrodes and the deformed Au balls, and (III) the contact resistance of the stitch bonds. Sixteen microchips were evaluated, showing a difference in the resistance of 0.19 Ω, before and after wire bonding (Fig. 4f and Supplementary Table S3). Therefore, the contact resistance of the realized around-the-edge metallic contacts is concluded to be negligible, demonstrating excellent electrical conductance of the contacts. In addition, to verify the absence of unwanted short circuits between the different electrodes after wire bonding, the resistances between the central bond pad and the two adjacent pads were measured. All the measured resistance values exceeded the measurement range of the instrument (*R* > 200 MΩ), confirming the correct insulation between different electrodes. Another sidewall trench design with a more complex geometry was also successfully realized and tested (Supplementary Figs. S1 and S 6). This alternative design provided a larger Au-to-Au contact area after bonding, although the difference in contact resistance could not be verified to be statistically significant.

Additional measurements to verify the electrical reliability of the edge contacts were conducted on randomly selected microchips that were either attached to a thermal release tape (three chips) or that were assembled and wire-bonded (four chips), respectively. All evaluated microchips demonstrated reliable operation at a current below 20 mA without any induced failure of the edge contacts. Currents of ~35 mA resulted in Joule heating of the microchips to a temperature of ~150 °C, as indicated by their detachment from the thermal released tape. Further increase of the current levels induced increasing heating of the thin-film Au resistors on the vertically wire-bonded microchips due to insufficient heat dissipation. Therefore, under the present configuration, the limiting factors for the current capacity are the over-heating of the thin-film resistors and the heat dissipation efficiency through the microchips rather than the ball bond contacts themselves. The demonstrated current capacities are compatible with, e.g., the targeted sensing applications, which typically involve currents in a range from nanoamperes to microamperes^5^.

Pull tests showed excellent mechanical robustness of the ball bonds performed on the sidewall trenches of the microchips. The edge ball bonds turned out to be the most robust parts of the wire bonds and more than seven times stronger than standard requirements^45^. As the weakest points of the wire bonds were the stitch bonds (see Section 3.2, Supplementary Information), pull tests were performed after glue fixation of the stitch bonds. In this situation, all the tested bonded Au wires were torn before any failure in the ball-to-trench interfaces occurred. The measured mean force when the Au wires broke was 175.4 mN, with a minimum force of 168.6 mN. These tests demonstrated the excellent bond strength of the ball bonds in the sidewalls of the microchips, which were the strongest parts of the Au connections.

**Fig. 5:**
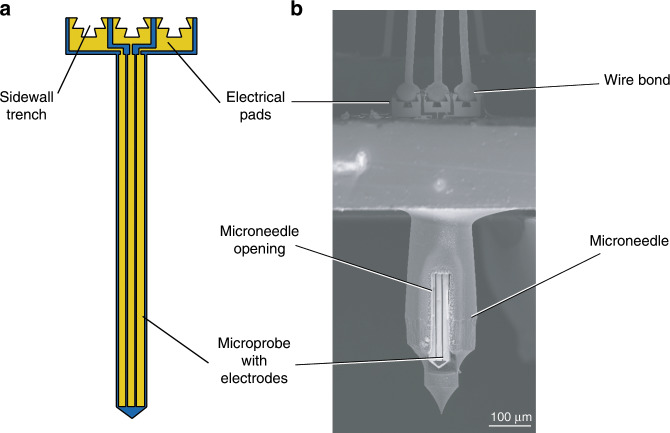
Edge wire bonding for microsensor applications. **a** Illustration of an edge wire bonding demonstrator representing a three-electrode sensor microprobe that can fit inside the lumen of a hollow silicon microneedle. **b** SEM image of the demonstrator microprobe placed inside a microneedle lumen and electrically connected using edge wire bonding.

Finally, to demonstrate the utility of this contacting approach for applications consisting of vertically integrated microchips that are extremely fragile, we performed edge wire bonding on “T”-shaped microprobes inserted in the lumen of hollow silicon microneedles. These microprobes have the same thickness and length as the previously presented microchips, but a narrower body (a 65 µm-wide probe) and a narrower 280 µm-wide top part of the “T” shape (Fig. 5a and Supplementary Fig. S1c). The overhanging top part of the microchips is proportionally wider than for the geometry in Fig. 1b, to provide enough space for the wire bonds. The bonding force for forming the ball bond was tuned accordingly to 250–400 mN for the smaller used FABs (70 µm diameter) to avoid damage of the fragile Si probes. In particular, such microprobes represent miniaturized sensors, similar to the enzymatic biosensors developed in a previous work and designed to fit in a microneedle lumen^5^. Figure 5b shows a non-functionalized demonstrator of a “T”-shaped three-electrode microprobe inserted in the lumen of a microneedle and electrically contacted using edge wire bonding to create interconnections between the electrodes on the microchip and the device package. In particular, such sensing microprobes require an assembly and electrical contacting method in which all the previously mentioned requirements and restrictions simultaneously apply. They consist of sub-millimeter-sized microchips, too tiny and fragile to be mechanically handled, which need to be assembled with a certain vertical orientation and might suffer the presence of materials that can either interfere with their operation or dramatically increase their fabrication complexity. Moreover, biosensors are extremely sensitive after the deposition of enzymatic membranes on the electrodes. In fact, although the silicon-based probes can be processed with standard microfabrication techniques, after the functionalization they cannot be exposed to solvent treatments and require room-temperature handling to preserve their functionality. Despite the fragility of such miniaturized devices, the microchips were intact after wire bonding, demonstrating the applicability and further miniaturization possibility of the proposed technology. Hence, this is an example in which the presented process could possibly be of use even with a sparse array of receiving holes, because of the impracticality to mechanically handle and contact such tiny and fragile devices.

## Conclusions

A novel approach for heterogeneous 3D vertical assembly and electrical contacting of microchips was proposed and evaluated. The microchips and the receiving substrate were fabricated separately, enabling the use of standard materials and in-plane microfabrication techniques, followed by the integration of the two components as the last step. The microchips were manipulated by a dynamic magnetic field and directionally assembled into matching holes in the receiving substrate, independently of the microchip aspect ratios. Assembly experiments suggest that the process can be applied to large microchip arrays. Future work on the front/back orientation control of the microchips during the magnetic assembly phase and analytical modeling of the magnetic-field-assisted assembly process could further widen the applicability and generality of the proposed method. Electrical connections between the vertically assembled microchips and the receiving substrate were created using a commercial wire bonder. The realized wire bonds showed negligible contact resistances (<0.2 Ω) and excellent bond strengths, well above wire bonding standard requirements (>168 mN in pull tests). The presented method enables directional vertical assembly without direct contact and electrical interconnecting of millimeter and sub-millimeter microchips at room temperature, without using solvents or exotic materials for the microchip fabrication. Thus, this method can be useful for 3D assembly and packaging of various microsystems, such as biosensors, in which the microfabricated sensing elements may need to be moved out of the initial fabrication plane and vertically assembled in a specific substrate to fulfill their function.

## Materials and methods

### Fabrication process

**Fig. 6:**
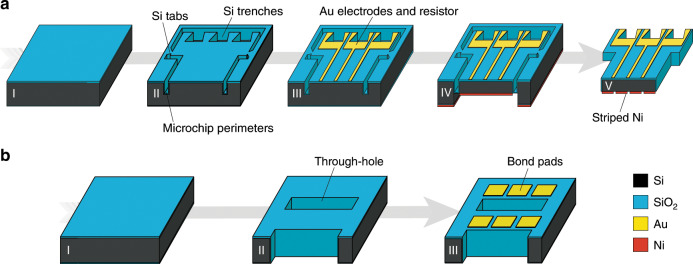
Illustration of the microfabrication process flow. **a** Microchip fabrication. The silicon wafer is thermally oxidized (**a**–**I**); trenches defining the microchips’ perimeters are formed with Si DRIE and a second thermal oxidation step is performed to electrically insulate the microchip sidewalls (**a**–**II**); Au electrodes and resistor lines are deposited using e-beam evaporation (**a**–**III**); Si DRIE is performed on the backside to define the microchip thickness; a nickel layer is deposited on the backside (**a**–**IV**) and laser patterned; microchips are released by breaking the silicon tabs along their perimeters (**a**–**V**). **b** Receiving substrate fabrication. The silicon wafer is thermally oxidized (**b**–**I**); through-holes matching the microchip geometries are formed using Si DRIE and then the wafer is again thermally oxidized (**b**–**II**); Au bond pads are deposited on the wafer frontside of the wafer, close to every hole (**b**–**III**). For the color code, please refer to the online version.

The fabrication process flows of the microchips and the receiving substrate are schematically illustrated in Fig. 6a, b, respectively. To realize the microchips, a 100 mm-diameter, 300 µm-thick Si wafer was used. The wafer was thermally oxidized and the SiO_2_ layer was used as hard mask in the following steps (Fig. 6a–I). Silicon trenches (70 µm-deep) were etched in the wafer frontside using deep reactive ion etching (DRIE), after oxide patterning, to form both the perimeters of the microchips and the sidewall trenches for wire bonding. Four small silicon tabs were kept intact along the perimeter, to mechanically connect the microchips to the wafer and provide sufficient robustness for the remaining fabrication steps (Supplementary Fig. S2e). A second thermal oxidation was performed to ensure electrical insulation of the silicon trenches around the microchips (Fig. 6a–II). Au electrodes and resistors (100 nm-thick) were then deposited by evaporation, over a 20 nm-thick Ti adhesion layer, and patterned using lift-off photolithography (Fig. 6a–III). Then, a backside lithographic step was performed and the SiO_2_ was selectively etched using RIE, followed by another Si DRIE step, to form recesses matching the frontside structures and define the final microchip thickness (70 µm). Finally, a 3 µm-thick nickel layer was deposited via sputtering on the backside of the wafer (Fig. 6a–IV). A femtosecond laser (Spirit, Spectra-Physics, MKS Instruments, USA) was used to pattern the nickel layer to assist magnetic assembly and to release the microchips from the wafer, by ablating the four supporting Si tabs and the remaining materials present on the bottom SiO_2_ layer surrounding the microchip perimeter (Fig. 6a–V). The femtosecond laser parameter used for the nickel patterning were as follows: *λ* = 520 nm, pulse repetition rate = 4 kHz, movement speed = 400 µm/s, 0.25 NA for the focusing objective, airflow during operation to remove micro-debris, 20 µm of line pitch, and 1.88 mW average power before the objective. For releasing the microchip by cracking the silicon tabs, the parameters were as follow: *λ* = 520 nm, pulse repetition rate = 1 kHz, movement speed ~2 µm/s, and 16.8 mW average power before the objective. The tabs ablation process was performed under water to avoid re-deposition of small particles and heat accumulation, and to simplify the collection of the released microchips. Alternatively, microchips were also released in parallel in water using ultra-sonication. Ultra-sonication was able to crack the oxide layer and, at increased power, also to selectively break the Si tabs at locations where the tabs were previously indented by the laser machining. Depending on the fragility of the realized microchips and the sensitivity of the device surfaces of the specific application, the direct laser release could be preferable as compared with the faster but less gentle ultra-sonication method. In addition, if desirable, the use of laser ablation could be substituted by, e.g., using a shadow mask or a dry-film resist for the patterning of the nickel layer, and by using dicing for the microchip release.

To realize the receiving substrate, a 100 mm-diameter, 500 µm-thick Si wafer was thermally oxidized to create a top insulation layer (Fig. 6b–I). Then, the SiO_2_ layer was selectively etched by RIE, and through-holes were formed using DRIE. The through-silicon holes were then thermally oxidized to avoid unwanted electrical contacts between the receiving substrate and the assembled microchips (Fig. 6b–II). Finally, to create the three bond pads near each hole on the frontside of the wafer, a 60 nm/300 nm-thick TiW/Au layer was sputtered on the substrate and patterned using lift-off photolithography (Fig. 6b–III).

### Magnetic assembly

Magnetic assembly experiments were conducted on the fabricated 500 µm-thick silicon receiving substrate with rectangular through-holes. The receiving substrate was placed at the bottom of a glass petri dish for the duration of the experiments. The microchips with the striped nickel coating on the backside were spread over the receiving substrate. A permanent neodymium magnet (N48, nominal residual magnetism of 1.37–1.42 T, rod-shaped with a diameter of 6 mm) was then placed under the petri dish to induce vertical lifting of the microchips. The receiving holes were located 1 mm apart from each other (center-to-center) and arranged into separate arrays. The holes had a 25 µm gap on each side between the sidewall of the holes and the assembled microchips. The arrays of holes used in the magnetic assembly experiments contained one, two, three, four, or six holes in line. The holes in each array were arranged in a line with short sides of the rectangular holes facing each other. The reason to choose in-line arrays was to guarantee a better statistical coherence, as the group of microchips was confined by the magnetic field in an area smaller than the inter-row distance (as shown in Supplementary Video 3). As a consequence, with the specific magnet used and the magnet motion characteristics described below, a magnetic sweep on an in-line array (e.g., 1 × 4 holes) can statistically be considered equivalent to a sweep on the different lines of the two-dimensional array with the same total number of holes (e.g., 2 × 2 holes) moving back and forth. For each array size, the assembly experiment was repeated 50 times, using the same number and type of microchips (39 microchips, 500 µm top width, 25 µm gap between the microchip, and the matching receiving hole). The magnet was manually moved back and forth along the long edges of the rectangular holes. After each sweep of the magnet over the array, the array was visually observed for any newly filled holes. Disassembly of an already filled hole was a very rare event only observed a few times during the whole experimental process. Each array was completely filled 50 times, while the number of sweeps to fill each hole in an array was recorded.

Data from the assembly experiments was collected and processed to represent the number of magnet sweeps needed to fill the next hole after a previous hole was filled. Each step of filling of an array, being it the filling of the first hole or filling the last hole of the array, followed an exponential probability distribution and was modeled accordingly (Supplementary Fig. S3). The fitting parameter for the exponential distribution is the mean number of magnet sweeps *m* to fill an individual hole. Thus, the probability density function *f*(*x*) of such a distribution can be expressed using the following equation:

1 $$f\left( x \right) = \frac{1}{m}e^{ - \frac{x}{m}}$$

where *x* stands for the variable, i.e., the number of magnet sweeps. A hypoexponential distribution represents a series of exponentially distributed events and is thus suitable for modeling the stochastic process of completely filling an array. The information gathered from the exponential distributions was used to create the corresponding hypoexponential distributions. The resulting probability density function *f*(*x*) of the modeled hypoexponential distribution can be expressed using the following equation:

2 $$f\left( x \right) = \mathop {\sum }\limits_{i = 1}^k \frac{1}{{m_i}}e^{ - \frac{x}{{m_i}}}\left( {\mathop {\prod }\limits_{j = 1,j \ne i}^k \frac{{\frac{1}{{m_j}}}}{{\frac{1}{{m_j}} - \frac{1}{{m_i}}}}} \right)$$

where *m*_*i*_ represents the mean number of magnet sweeps to fill the *i*^th^ hole in the studied array of *k* holes. These hypoexponential distributions matched well with the data representing the probability distributions of completely filling holes arrays of different sizes (Supplementary Fig. S4). The exponential distribution from Eq. (1) was fitted directly to the measured data points in the statistical analysis of the assembly process (e.g., Fig. 3a). It should be noted that these fits are not based on the histograms, which only aid the visualization of the distribution of the measured values. The bin widths of the histograms were selected according to the range over which the measured 50 data points extended, with the aim of not having too wide bins hiding the distribution shape nor too narrow bins amplifying the noise in the data.

### Wire bonding

After the assembly, the microchips were fixated by applying small droplets of glue (Super glue, Loctite-Henkel, Germany) on the sides of the microchips, to assist reliable wire bonding. In a setting for volume manufacturing, this could be performed using an automatic dispensing tool, precisely applying a defined amount of glue at the supporting overhanging parts of the microchips. The glue may remain (as in the case of the chips used for pull tests) or be dissolved with acetone after the microchips have been connected (as in Fig. 1d), depending on the requirements of the application. Wire bonding was performed using a commercial wire bonder (ESEC 3100+, ESEC, Ltd, Switzerland) at a substrate temperature of 30 °C, using an Au wire diameter of 25 µm, a FAB diameter of 90 µm, an average ball bond force of 575 mN (100 ms bond time, no ultrasonic energy applied), and a stitch bond force of 500 mN (50 ms, 30% ultrasonic energy applied). The bonds were performed with a certain offset towards the frontside electrodes, to enhance the contact area. However, if this offset value was too small (as defined in Fig. 4b), it could result in weak mechanical fixation of the Au balls inside the trenches. Experiments showed that with 90 µm-diameter FABs and 60 µm-wide trenches, an offset of 10–20 µm is a suitable range to get reliable bonding. Additional details and parameters regarding FAB formation, ball bonds, and stitch bonds used in the experiments with the different microchip designs are reported in Supplementary Table S2. Metallic lines are incorporated on the microchips as simple resistors between the left and the right frontside electrodes, to help evaluate the quality of the metallic contacts. The central electrode is electrically isolated from the other two electrodes, to verify proper insulation, as illustrated in Fig. 1b. The resistance values of the device resistors before and after wire bonding were measured via microprobes using a four-wire configuration (2450 SourceMeter SMU, Keithley Instruments, USA). Pull tests were performed using a commercial pull tester (Dage 4000Plus, Inseto Ltd, UK). Under normal conditions, all the broken points occurred at the stitch bonds on the receiving substrate and not on the ball bonds on the microchip side (see Section 3.2, Supplementary Information for more details). Therefore, to evaluate the ball-to-trench bond strength at the microchip side, a new set of wire bonds were tested after the stitch bonds on the receiving substrate were fixated by epoxy glue (Quick-epoxy, Biltema, Sweden). In this case, all the tested bonded Au wires were torn before occurrence of any ball-to-trench bond failure. Twelve pairs of wire bonds were evaluated in the first set of standard pull tests, whereas six wire bonds were evaluated in the second set of pull tests, where the stitch bonds were fixated by epoxy glue.

## Supplementary information

Supplementary video 1

Supplementary video 2

Supplementary video 3

Supplementary video 4

Supplementary information

Five sheets of datasets used for the paper.
