# Supplementary material for: Vertical integration of microchips by magnetic assembly and edge wire bonding
Source: Microsyst Nanoeng. 2020 Feb 24;6:12. doi: 10.1038/s41378-019-0126-6 (PMC8433299; doi:10.1038/s41378-019-0126-6)
Supplement: Supplementary file 5 — Supplementary information [file 41378_2019_126_MOESM5_ESM.pdf]

Vertical integration of microchips by magnetic assembly and edge wire bonding

Federico Ribet, Xiaojing Wang, Miku Laakso, Simone Pagliano, Frank Niklaus,
Niclas Roxhed, Göran Stemme

SUPPLEMENTARY INFORMATION

1. Microchip designs and size variations

After preliminary testing of several trench geometries for edge wire bonding, two silicon trench designs were characterized and proved reliable, both in terms of contact resistance and mechanical fixation. In particular, while several combinations of shapes and dimensions were evaluated, two trench geometries provided the simplest and most reliable solutions. The first geometry is based on a single trapezoid trench, as illustrated in Fig. S1a (design A, presented in the main article). The second design, with a double-trapezoid trench geometry (Fig. S1b, design B), aimed at increasing the potential metallic contact area between the deformed Au free-air-balls (FABs) and the frontside electrodes. The important dimensions of the two designs are listed in Table S1, and are defined according to the illustrations in Fig. S1. Fig. S1c illustrates the geometry of the sensing microprobe demonstrator, a possible targeted application that requires the microprobe to be reliably vertically assembled and contacted. The microprobe demonstrator featured top trench geometries similar to design B, but with a top sidewall trench opening width of 50 µm only. The bonded FABs were accordingly scaled down to 70 µm in diameter. The thickness of all presented microchips was 70 ± 5 µm.

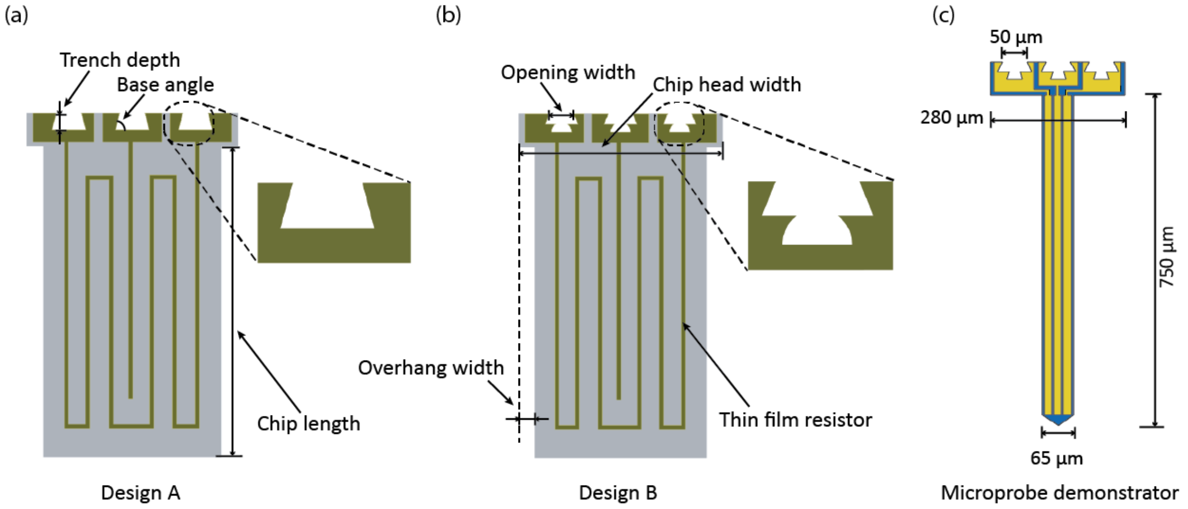

Figure S1. (a, b) Illustration of two different microchip designs (design A and design B, respectively) with different sidewall trench geometries for ball bonds and definitions of the parameters reported in Table S1. Design A has a single trapezoid geometry, whilst design B has a double trapezoid shape for enhanced metallic contact area between the deformed Au FABs and the frontside electrodes. (c) Geometrical details of the microprobe demonstrator, fitting inside the lumen of a hollow silicon microneedle. The top part of the "T" shape is narrower, close to the miniaturization limit to perform reliable wire bonding.

Table S1. Key dimensions and variations of the presented microchip designs (excluding microsensor demonstrator).

Design		Dimensions of features		
		Opening width (µm)	Trench depth (µm)	Base angle (degrees)
A	Trench	60	40	76
B	Top trench	60	25	68
	Bottom trench	30	20	63
Other parameters		Overhang width (µm)	Chip length (µm)	
		40	750	
		Chip thickness (µm)	Chip head width (µm)	
		70 ± 5	330 / 500 / 1000 / 1500	

2. Magnetic assembly

2.1 Nickel patterning and microchip size variations

Microchips with different aspect ratios (microchip length/width, with the microchip body length fixed to 750 µm) were tested to verify that the lifting direction can be controlled independently of the microchip size or aspect ratio, after Ni patterning. Fig. S2a-d show the backside of the four different microchips, with different widths, after Ni striping. Fig. S2e shows the silicon tabs physically connecting each microchip with the silicon wafer, left during the dry etching step defining the microchips' perimeters, in order to preserve mechanical stability during the following microfabrication steps.

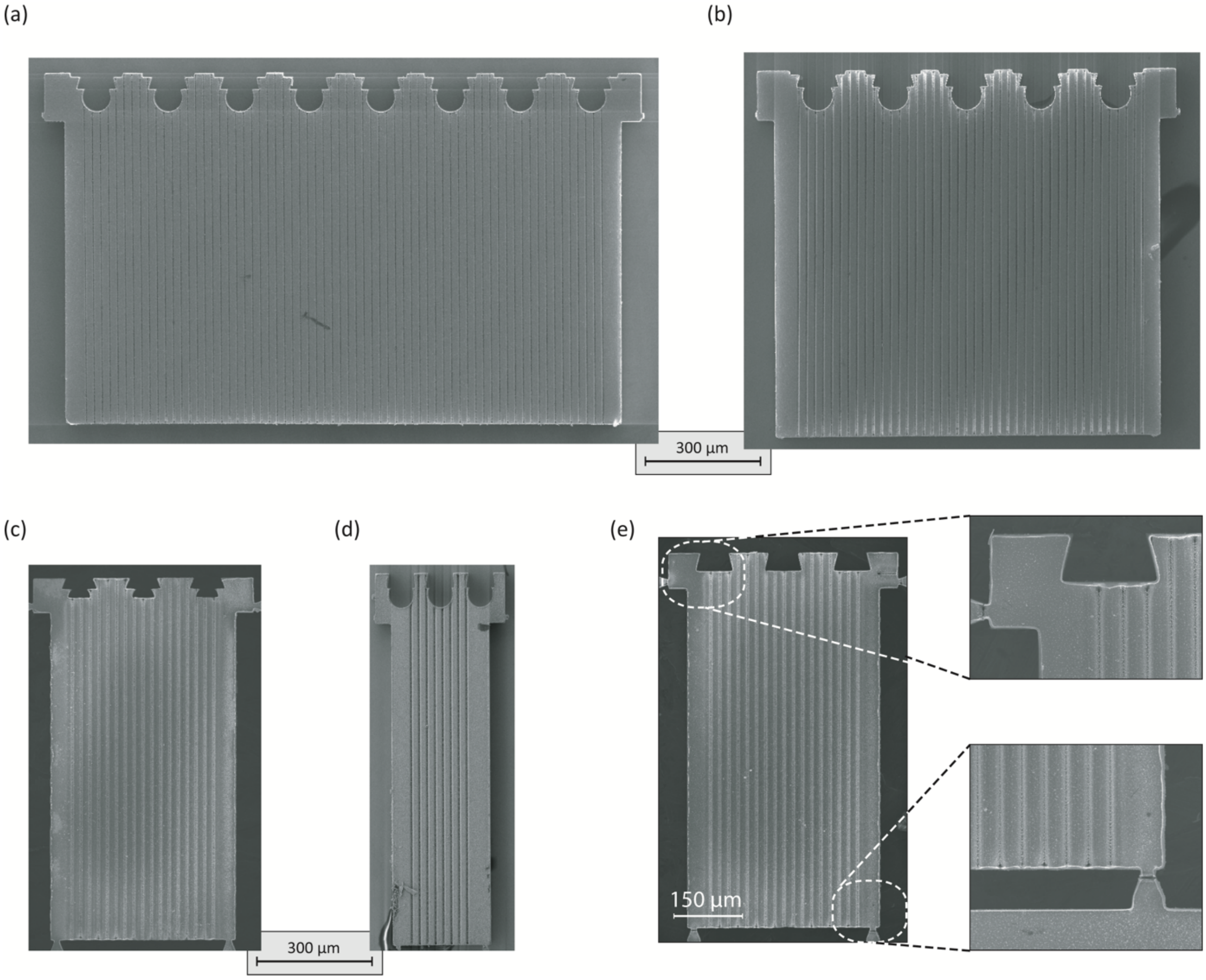

Figure S2. SEM images of the backside of the different microchip geometries that were studied, having the same length (750 µm) but different widths. The chip aspect ratios range from 2.3 to 0.5 (length/width). The pictures show: (a) 1500-µm-wide chip, (b) 1000-µm-wide chip, (c) 500-µm-wide chip, and (d) 330-µm-wide chip, respectively. (e) SEM picture of a 500-µm-wide microchip with enlarged views of the Ni striping, and of the silicon tabs that were used during the microfabrication process to keep each microchip attached to the silicon wafer and eventually cracked to release the microchips.

2.2 Probability distribution of magnetically assembling individual holes in an array

The magnetic assembly of microchips into the receiving holes was statistically investigated for five different array sizes (one, two, three, four, and six-hole arrays). For each studied array size, the assembly test was repeated 50 times using 39 500-µm-wide microchips. Each progressive step of filling an array had an exponential distribution. A typical example is shown in Fig. S3, where consecutive steps of filling the 6-hole array are presented with their fitted exponential distributions.

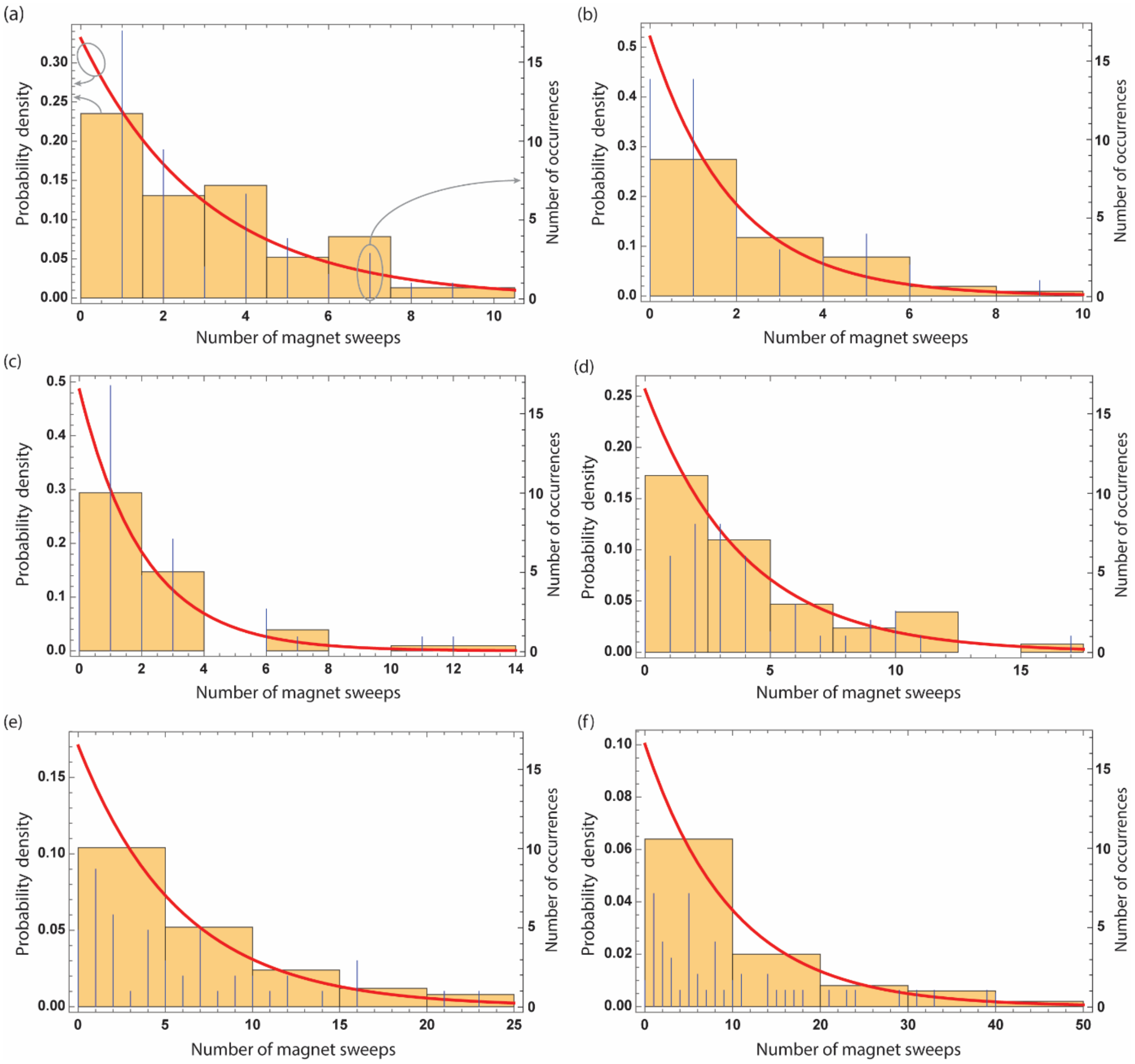

Figure S3. The number of magnet sweeps needed to fill the six-hole array. (a-f) Sub-figures show the magnet sweeps needed to fill the array starting from the first hole and ending to the last hole to be filled in the array. 39 microchips were used in the assembly process. Each magnetic assembly event is represented by a blue vertical line above the horizontal axis. If the same number of sweeps was required on multiple occasions, the height of the line was increased, which is represented on the vertical axis on the right side of the figures. The exponential probability-density-distribution curves from Equation (1) were fitted directly to the measured datapoints. The histograms aid the visualization of the distribution of the measured values. All the sequential probability densities for filling a hole follow an exponential dependency on the number of magnet sweeps.

2.3 Completely filling arrays of different sizes

The probability-density distributions of completely filling arrays of different sizes can be modeled as hypoexponential distributions (Fig. S4). The hypoexponential distributions were created using the exponential distributions fitted to each consecutive step of filling an array of holes. As expected, the mode of a hypoexponential distribution shifts right with an increasing array size (Fig. S4f).

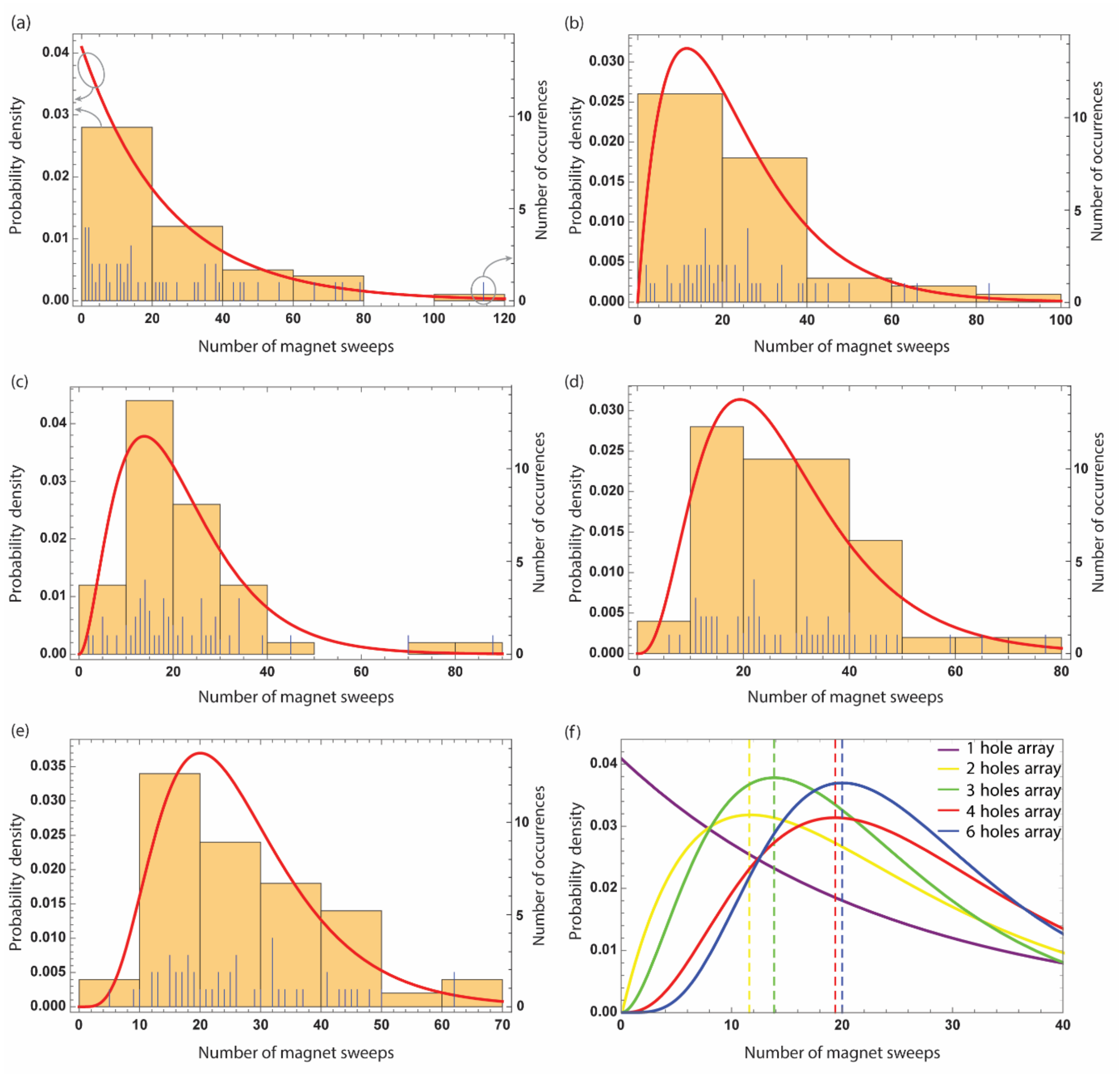

Figure S4. The number of magnet sweeps needed to completely fill an array. (a-e) Data collected from different array sizes having one, two, three, four, or six holes. Each magnetic assembly event is represented by a blue vertical line above the horizontal axis. If the same number of sweeps was required on multiple occasions, the height of the line was increased, which is represented on the vertical axis on the right side of the figures. The hypo-exponential probability-density-distribution curves from Equation (2) were fitted directly to the measured datapoints. The histograms aid the visualization of the distribution of the measured values. (f) The fitted probability-density functions from sub-plots 'a' to 'e' are presented for easier comparison. The vertical dashed lines indicate the mode values of the fitted probability-density functions.

2.4 Effects of gap size, microchip number and rotational freedom on assembly efficiency

In order to study the effects of rotational freedom, we performed assembly tests using different microchip widths and different widths of gaps between a microchip and the side walls of a receiving hole. Fig. S5 shows the effects of different chip widths, gap sizes (tolerance between the microchip and the matching receiving hole) and number of microchips used for the assembly test on the resulting assembly efficiency, in accordance with the different degrees of rotational freedom. Fig. S5f shows that larger gap sizes and narrower chips tend to increase the assembly efficiency. A larger number of microchips can increase the assembly efficiency as well. However, this latter effect is less evident than the previous two, probably because, regardless of the number of microchips available, only a limited number of them can move over a single hole in a single magnet sweep. This effect would likely be evident when the total number of chips approaches or equals the number of holes. The effects on the assembly efficiency of both gap size and microchip width can be explained by looking at the range of rotational angles of the microchips that allows them to drop into the receiving holes (Fig. S5g). By using the rotational freedom, assembly speeds for different microchip and receiving hole size variations can be forecasted. By using the available rotational angles as a scaling factor for the mean number of magnet sweeps, forecasts for the probability density distributions of 500-µm-wide microchips with 10 µm gap and 330-µm-wide microchips with 20 µm gap have been made (Fig. S5f), based on the assembly data from 500-µm-wide microchips with 25 µm gap. These forecasts are in accordance with the measured data (Fig. S5f). This indicates that the range of allowable rotational angles of the microchips to fit in the receiving holes has a major effect on the assembly efficiency. A circular receiving hole would therefore significantly increase the assembly efficiency. Faster assembly into circular holes might be preferable in certain applications and it would be compatible with the electrical contacting technique presented hereafter. However, for applications where the rotational positions of assembled microchips are important, rectangular holes serve a role for aligning the microchips and, additionally, can save area on the receiving substrate.

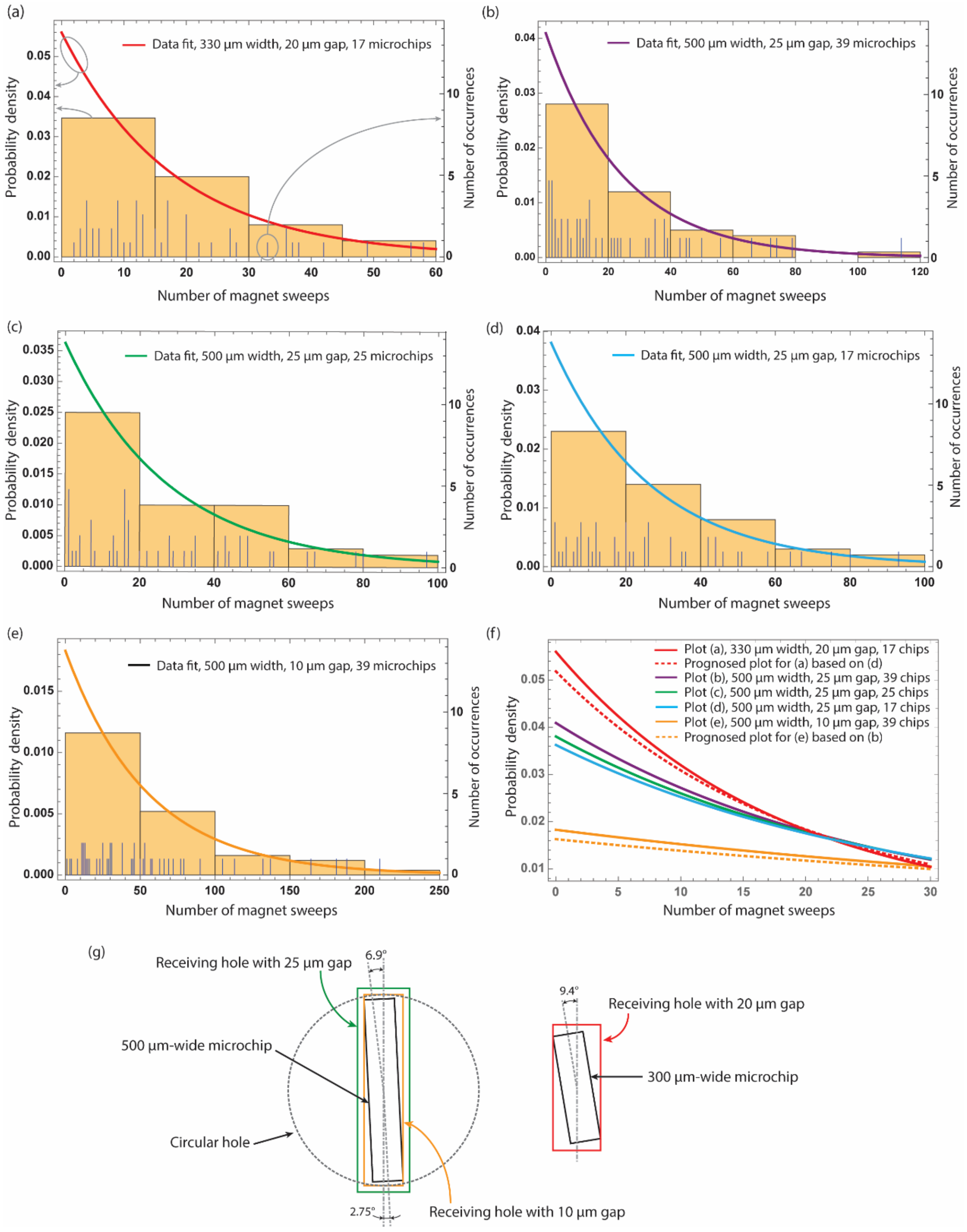

Figure S5. (a-e) Probability density curves for five evaluated groups featuring different microchip/gap sizes and number of microchips used for the assembly test, as specified in each plot. Each magnetic assembly event is represented by a blue vertical line above the horizontal axis. If the same number of sweeps was required on multiple occasions, the height of the line was increased, which is represented on the vertical axis on the right side of the figures. The histograms aid the visualization of the distribution of the measured values. (f) Probability density distributions of the five evaluated groups plotted together, combined with two prognosed probability distributions (dotted lines). (g) In-scale illustration of rotational angles available for microchips with different widths fitting inside the matching receiving holes, with different gap sizes.

3. Wire bonding

3.1 Wire bonding parameters

Table S2 reports the parameters to perform wire bonding on microchips with design A and B. The minimum force needed for placing a ball bond is larger for design B, since more deformation of the FAB into the trenches has to be induced for reliable mechanical fixation. Fig. S6 shows the edge wire bonding results for design B from different viewing angles.

Table S2. Wire bond process parameters.

Parameters		Design A	Design B
Wire diameter (µm)		25	
Au FAB diameter (µm)		90	
Substrate temperature (°C)		30	
Ball bond	Force (mN)	400-750	550-750
	Time (ms)	100	
	Ultrasound level (%)	0	
Stich bond	Force (mN)	500	
	Time (ms)	50	
	Ultrasound level (%)	30	
Loop height (µm)		350	

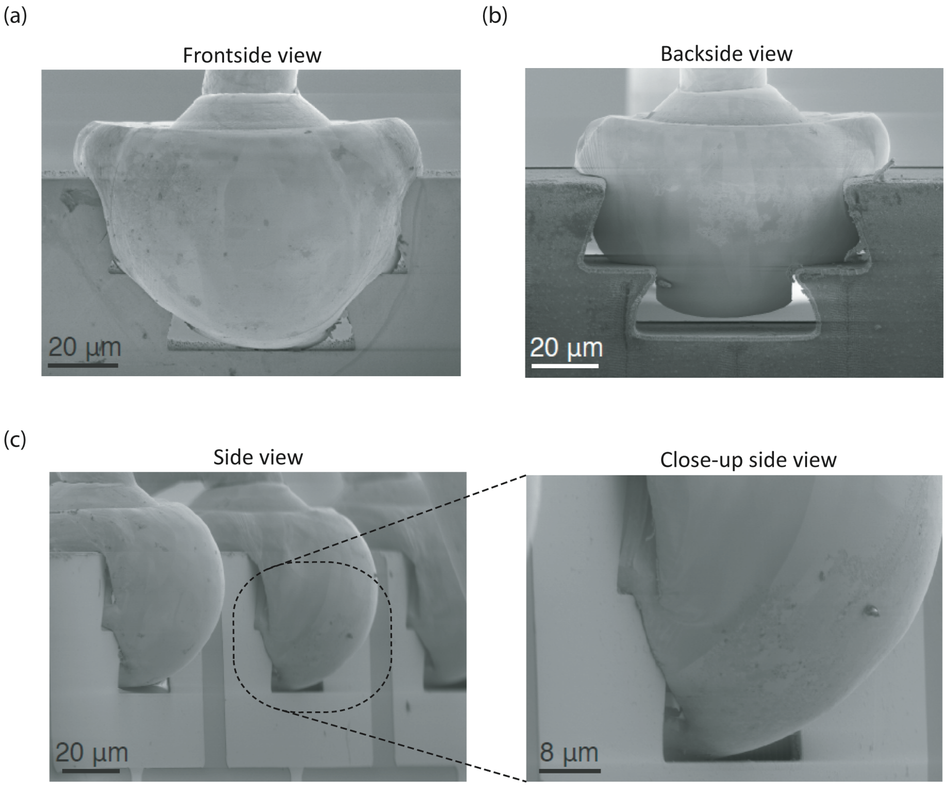

Figure S6. (a, b, c, d) Close-up SEM images, from different angles, of the Au ball bond deformation inside and around the silicon trenches of the microchips with design B. (a) Frontside view of the Au-to-Au contact area between a deformed Au FAB and the electrode. (b) Backside view of a ball bond showing the wedging of the ball bond inside the trench. (c) Partial side view and close-up view of the over-the-edge Au-to-Au contact area location. With this design, the area of the Au-to-Au contact is further increased to obtain reliable electrical contact.

3.2 Pull tests

The measured mean pulling strength for breaking the wire bonds in any location was 92.1 mN, with a minimum measured pulling force for breaking a wire bond of 63.7 mN. In fact, all the broken points occurred at the stitch bonds on the receiving substrate and not on the ball bonds on the microchip side. These values were nonetheless almost four times larger than 23.5 mN required in military standard, showing more than sufficient mechanical strength of the wire bonds. Even after stich bond fixation by epoxy glue, all the tested bonded Au wires were torn before occurrence of any ball-to-trench bond failure.

3.3 Electrical characterization

The contact resistance was also studied for both designs, by comparing the measured resistance of the thin-film resistors before and after bonding. The average increase in the resistance values for design A and B are 0.19 Ω and 0.08 Ω, respectively, which include the contribution from the contact resistances of the edge ball bonds. The average increase in resistance is lower for design B than design A, even though both values are significantly smaller than the standard errors of the sample mean. Therefore, the contact resistances of the edge ball bonds for both microchip designs are negligible. The main advantage of design B over design A is related to the increased Au-to-Au bond area, which could be seen in the SEM inspection (c.f. Fig. 4c versus Fig. S6c), potentially resulting in improved reliability in a production environment. Table S3 summarizes the results of the resistance measurements.

Table S3. Measured resistance values of the microchip resistors before and after wire bonding (n = 16).

**Design**	**Before wire bonding** (on the microchip frontside pads)		**After wire bonding** (on the receiving substrate pads)		**Mean variation** ΔR (Ω)
	Mean (Ω)	Standard error of the sample mean (Ω)	Mean (Ω)	Standard error of the sample mean (Ω)	
A	121.31	0.52	121.50	0.50	+0,19
B	116.78	0.44	116.86	0.52	+0,08
